# Supplementary material for: Identification and functional analysis of non-coding regulatory small RNA FenSr3 in Bacillus amyloliquefaciens LPB-18
Source: PeerJ. 2023 May 15;11:e15236. doi: 10.7717/peerj.15236 (PMC10194069; doi:10.7717/peerj.15236)
Supplement: Supplemental Information 4 [file peerj-11-15236-s004.zip › KO/CK-vs-T1_map/map05332.html]

KEGG PATHWAY: Graft-versus-host disease - Reference pathway


|  |  |
| --- | --- |
| **Graft-versus-host disease - Reference pathway** |  |

[
Pathway menu
| Organism menu
| Pathway entry
| Show description
| User data mapping
]

Graft-versus-host disease (GVHD) is a lethal complication of allogeneic hematopoietic stem cell transplantation (HSCT) where immunocompetent donor T cells attack the genetically disparate host cells. GVHD pathophysiology can be summerized in a three-step process. Step 1 involves the development of an inflammatory milieu resulting from damage in the host tissues induced by the preparative chemotherapy or radiotherapy regimen. Damaged tissues secrete inflammatory cytokines, including interleukin 1 (IL-1), and tumor necrosis factor (TNF-alpha ). During step 2, antigen-presenting cells (APCs) trigger the activation of donor-derived T cells, which induce further T-cell expansion, induce cytotoxic T lymphocytes (CTL) and natural killer (NK) cells responses and prime additional mononuclear phagocytes to produce TNF-alpha and IL-1. Also, nitric oxide (NO) is produced by activated macrophages, and it may contribute to the tissue damage seen during step 3. During step 3, the effector phase, activated CTL and NK cells mediate cytotoxicity against target host cells through Fas-Fas ligand interactions and perforin-granzyme B.

|  |  |  |
| --- | --- | --- |
| Reference pathway | 184% 150% 122% 100% 82% 67% 55% | 图片下载 |
